# Glycemic Status and Glycemic Variability Related to Mortality and Morbidity in Critically III Diabetic and Non-Diabetic Patients: A Prospective Observational Study

**DOI:** 10.3390/clinpract16080145

**Published:** 2026-08-06

**Authors:** Mădălina Diana Fehér, Codrin Dan Nicolae Ilea, Cosmin Mihai Vesa, Alina Cristiana Venter, Simona Daciana Birsan, Timea Claudia Ghitea, Rareș Cristian Daina, László Fehér, Cristian Marius Daina

**Affiliations:** 1 Doctoral School of Biomedical Sciences, Faculty of Medicine and Pharmacy, University of Oradea, 1 December Sq., 410081 Oradea, Romania; diana_daina98@yahoo.com (M.D.F.); feher.laszlo01@yahoo.com (L.F.); 2 Department of Psycho-Neurosciences and Recovery, Faculty of Medicine and Pharmacy, University of Oradea, 1 December Sq., 410081 Oradea, Romania; codrin.ilea@csud.uoradea.ro (C.D.N.I.); simona2372@yahoo.com (S.D.B.); cristi_daina@yahoo.co.uk (C.M.D.); 3 Department of Preclinical Disciplines, Faculty of Medicine and Pharmacy, University of Oradea, 1 December Sq., 410081 Oradea, Romania; v_cosmin_15@yahoo.com; 4 Department of Morphological Disciplines, Faculty of Medicine and Pharmacy, University of Oradea, 410073 Oradea, Romania; aventer@uoradea.ro; 5 Pharmacy Department, Faculty of Medicine and Pharmacy, University of Oradea, 1 December Sq., 410081 Oradea, Romania; 6 Study Program, Medicine in English Language, Faculty of Medicine and Pharmacy, University of Oradea, 1 December Sq., 410081 Oradea, Romania; rares.daina06@gmail.com

**Keywords:** critical illness, intensive care unit, hyperglycemia, stress hyperglycemia, diabetes mellitus, glycemic variability, coefficient of variation, hypoglycemia, mortality, ICU morbidity

## Abstract

**Background:** Glycemic dysregulation is frequent in critically ill patients and may influence prognosis regardless of pre-existing diabetes status. This study aimed to evaluate the impact of glycemic status and glycemic variability on mortality and morbidity in diabetic and non-diabetic patients admitted to the intensive care unit (ICU). **Methods:** This prospective observational study included 244 critically ill patients. Demographic, clinical, biological, and glycemic data were collected during ICU hospitalization. Glycemic parameters included admission glucose, mean glucose, glucose standard deviation, coefficient of glycemic variability, hyperglycemia > 140 mg/dL, hyperglycemia > 180 mg/dL, hypoglycemia < 70 mg/dL, and intravenous insulin requirement. The primary outcome was in-hospital mortality. **Results:** In-hospital mortality was 58.6%. Non-survivors had significantly higher admission glucose, mean glucose, glycemic standard deviation, and coefficient of glycemic variability compared with survivors. Hyperglycemia > 140 mg/dL, hyperglycemia > 180 mg/dL, poor glycemic control, and intravenous insulin therapy were significantly associated with mortality. Patients with high glycemic variability, defined as coefficient of variation (CV) > 36%, had higher mortality than those with lower variability. In multivariable analysis, mean glucose remained the most robust glycemic predictor associated with mortality, while glycemic variability retained prognostic relevance in alternative models. **Conclusions:** Glycemic dysregulation was common and clinically relevant in critically ill diabetic and non-diabetic patients. Dynamic glucose monitoring, including mean glucose and glycemic variability, may improve risk stratification and support structured ICU glycemic management.

## 1. Introduction

Glycemic dysregulation is a frequent metabolic disturbance in critically ill patients and may occur both in individuals with pre-existing diabetes mellitus and in those without previously known diabetes. In the intensive care unit (ICU), acute illness is commonly accompanied by stress-related hyperglycemia, driven by inflammatory activation, counter-regulatory hormone release, insulin resistance, altered hepatic glucose production, organ dysfunction, and therapeutic interventions such as vasopressors, corticosteroids, nutritional support, and dextrose-containing fluids [1,2].

Hyperglycemia in critically ill patients has been associated with increased morbidity and mortality, but optimal glucose management remains complex. Earlier approaches favoring intensive glucose control were challenged by evidence showing increased hypoglycemia and mortality risk when very strict glycemic targets were pursued. As a result, current recommendations generally support moderate glycemic control in most critically ill patients, with insulin therapy usually initiated for persistent glucose values ≥ 180 mg/dL and a target range of 140–180 mg/dL after therapy initiation [3,4].

Beyond absolute glucose values, glycemic variability has emerged as an important marker of metabolic instability in ICU patients. Fluctuations in blood glucose may reflect dynamic changes in insulin sensitivity, nutritional intake, renal and hepatic function, inflammatory burden, infection, hemodynamic instability, and treatment intensity. Glycemic variability may also contribute to oxidative stress, endothelial dysfunction, immune dysregulation, and organ injury, suggesting that unstable glucose patterns may be clinically relevant even when mean glucose values appear acceptable [5,6].

Several studies have suggested that glycemic variability is associated with adverse outcomes in critically ill patients, including short-term mortality. However, the relative prognostic value of mean glucose, hyperglycemic thresholds, hypoglycemia, and the coefficient of glycemic variability remains incompletely defined, particularly when diabetic and non-diabetic critically ill patients are analyzed together. Moreover, in routine ICU practice, glucose management often focuses on isolated hyperglycemic values, whereas dynamic parameters such as mean glucose and glycemic variability are less frequently incorporated into risk assessment and therapeutic decision-making [7,8,9].

The distinction between pre-existing diabetes mellitus and stress hyperglycemia is also clinically important. Patients without known diabetes who develop hyperglycemia during critical illness may represent a specific high-risk phenotype, in which acute metabolic dysregulation reflects the severity of systemic stress. Conversely, patients with known diabetes may have chronic adaptation to higher glucose levels, making the prognostic interpretation of hyperglycemia different across metabolic subgroups. Therefore, evaluating glycemic status together with dynamic glucose parameters may improve risk stratification in critically ill patients. In the absence of glycated hemoglobin (HbA1c) or follow-up testing, distinguishing true stress hyperglycemia from previously unrecognized diabetes remains challenging in critically ill patients.

The primary objective of this study was to evaluate whether glycemic status and glycemic variability were independently associated with in-hospital mortality in critically ill patients. Secondary objectives included the evaluation of individual glycemic metrics (admission glucose, mean glucose, hyperglycemia > 140 mg/dL, hyperglycemia > 180 mg/dL, hypoglycemia, insulin therapy requirement) and their relationship with ICU morbidity.

## 2. Materials and Methods

### 2.1. Study Design and Setting

This was a prospective observational study conducted in the ICU of Clinical County Emergency Hospital of Oradea, between 1 February 2026 and 1 June 2026. The study included critically ill diabetic and non-diabetic patients admitted to the ICU during the study period. The main objective was to evaluate the association between glycemic status, glycemic variability, ICU morbidity, and in-hospital mortality.

### 2.2. Study Population

The final study cohort included 244 critically ill patients admitted to the ICU. Patients were included if they were hospitalized in the ICU and had available clinical data and repeated blood glucose measurements during hospitalization.

Patients were excluded if they had incomplete essential clinical data, insufficient glycemic monitoring for the calculation of glycemic variability, or missing outcome data. Patients were analyzed regardless of pre-existing diabetes status.

According to metabolic status, patients were classified into three groups: patients with known diabetes mellitus, patients with stress hyperglycemia, and patients without diabetes mellitus or stress hyperglycemia. Diabetes mellitus was defined based on previous medical history or documented diagnosis in the medical records. Patients without a previous diagnosis of diabetes who developed hyperglycemia during critical illness were classified as having stress hyperglycemia according to the clinical database classification. However, because HbA1c measurements were not systematically available, previously unrecognized diabetes could not be completely excluded in this subgroup.

### 2.3. Data Collection

Clinical, demographic, biological, and glycemic data were collected prospectively during ICU hospitalization. The following demographic variables were recorded: age, sex, and place of residence. Clinical variables included type of ICU admission, Glasgow Coma Scale (GCS) score, Sequential Organ Failure Assessment (SOFA) score when available, comorbidities, need for mechanical ventilation, need for inotropic support, need for hemodialysis, ICU complications, and length of hospitalization.

Unfortunately, complete SOFA scores were available only for a subset of patients, while APACHE II scores were not routinely collected in our ICU database. Therefore, these variables could not be included in the primary multivariable model. Missing values resulted from incomplete routine documentation during ICU admission. The pattern of missingness was not formally assessed.

Comorbidities included arterial hypertension, chronic ischemic heart disease, chronic heart failure, previous stroke, chronic kidney disease, liver disease, malignancy, and other relevant chronic conditions recorded in the medical charts.

Biological and clinical parameters included admission blood glucose, repeated glucose values during hospitalization, lactate, pH, sodium, potassium, leukocyte count, neutrophil count, lymphocyte count, platelet count, systolic blood pressure, and diastolic blood pressure, where available.

### 2.4. Glycemic Variables and Definitions

Glycemic assessment was based on admission blood glucose and repeated glucose measurements recorded during ICU hospitalization. For each patient, the following glycemic parameters were calculated:

• admission blood glucose;
• mean glucose during hospitalization;
• standard deviation of glucose values;
• coefficient of glycemic variability;
• presence of hyperglycemia > 140 mg/dL;
• presence of hyperglycemia > 180 mg/dL;
• presence of hypoglycemia < 70 mg/dL;
• need for intravenous insulin therapy;
• glycemic control status.

The coefficient of glycemic variability was calculated as follows:

Coefficient of variation (CV) (%) = glucose standard deviation/mean glucose × 100.

A coefficient of glycemic variability > 36% was used to define high glycemic variability. This threshold was selected because it is frequently used in clinical studies as a marker of unstable glycemic control and increased risk of adverse outcomes.

Patients without previously diagnosed diabetes who developed hyperglycemia during critical illness were classified as having stress hyperglycemia according to the clinical database. Hyperglycemia was defined as at least one blood glucose value >140 mg/dL. HbA1c measurements were not systematically available; therefore, previously undiagnosed diabetes could not be completely excluded. The classification was consistent with current ICU practice and American Diabetes Association (ADA) recommendations regarding stress-related hyperglycemia. Hypoglycemia was defined as at least one glucose value < 70 mg/dL.

### 2.5. Outcomes

The primary outcome was in-hospital mortality, defined as death occurring either during ICU hospitalization or after transfer from the ICU to the general ward.

Secondary outcomes included:

• ICU mortality;
• mortality after transfer to the general ward;
• ICU complications;
• need for mechanical ventilation;
• need for inotropic support;
• need for hemodialysis;
• length of hospitalization;
• poor glycemic control.

ICU morbidity was assessed using recorded complications and the need for organ support during hospitalization.

### 2.6. Statistical Analysis

Statistical analysis was performed using IBM SPSS Statistics, version 30. Continuous variables were tested for distribution and expressed as mean ± standard deviation or median with interquartile range, depending on data distribution. Categorical variables were expressed as absolute numbers and percentages.

Comparisons between groups were performed using Student’s *t*-test or Mann–Whitney U test for continuous variables and the Chi-square test or Fisher’s exact test for categorical variables. Comparisons among the three metabolic groups were performed using one-way ANOVA or the Kruskal–Wallis test for continuous variables and the Chi-square test for categorical variables.

Survival status was analyzed by comparing survivors and non-survivors. Additional analyses were performed according to glycemic status, hyperglycemia thresholds, hypoglycemia, and glycemic variability categories.

Multivariable logistic regression was used to identify as a higher risk of in-hospital mortality. Variables were selected based on clinical relevance and univariate associations. The main models included age, sex, diabetes status, type of ICU admission, presence of comorbidities, glycemic parameters, and hypoglycemia. Mean glucose and hyperglycemia > 180 mg/dL were analyzed in separate models to reduce overlap between continuous and categorical measures of glycemic burden.

Because mean glucose and CV are mathematically related, collinearity was considered during model construction. Separate multivariable models were therefore developed to avoid excessive overlap between continuous measures of glycemic burden and glycemic variability. Formal collinearity diagnostics were not performed.

All clinically relevant variables were initially considered. No automated forward or backward selection procedure was applied. The final models were specified a priori based on clinical relevance and avoidance of collinearity. Because the univariate analyses were considered exploratory and hypothesis-generating, adjustment for multiple comparisons was not performed.

Odds ratios (ORs) with 95% confidence intervals (CIs) were reported. Model performance was evaluated using the area under the receiver operating characteristic curve and overall classification accuracy. A *p*-value < 0.05 was considered statistically significant.

### 2.7. Ethical Considerations

The study was conducted in accordance with the principles of the Declaration of Helsinki and applicable national regulations regarding biomedical research. The study protocol was approved by the Ethics Committee of the University of Oradea (approval no. 65, 30 January 2026). Written informed consent was obtained from all participants or from their legally authorized representatives prior to study inclusion. Because this was an observational study based on prospectively collected clinical data, patient management was not modified by study participation. All data were anonymized before statistical analysis to ensure confidentiality.

## 3. Results

### 3.1. General, Clinical, and Glycemic Characteristics of the Study Cohort

The study included 244 critically ill patients, with a mean age of 67.35 ± 14.60 years; 160 (65.6%) were male. Known diabetes mellitus was present in 76 patients (31.1%), 9 patients (3.7%) were classified as having stress hyperglycemia, and 159 (65.2%) had neither condition. Comorbidities were recorded in 200 patients (82.0%), and most admissions were for medical conditions (76.2%). Mechanical ventilation was required in 227 patients (93.0%), while ICU complications occurred in 194 (79.5%).

The mean admission glucose was 154.64 ± 76.21 mg/dL, and the mean glucose during hospitalization was 136.05 ± 48.37 mg/dL. Hyperglycemia > 140 mg/dL occurred in 182 patients (74.6%), hyperglycemia > 180 mg/dL in 67 (27.5%), and hypoglycemia < 70 mg/dL in 64 (26.2%).

**Figure 1:**
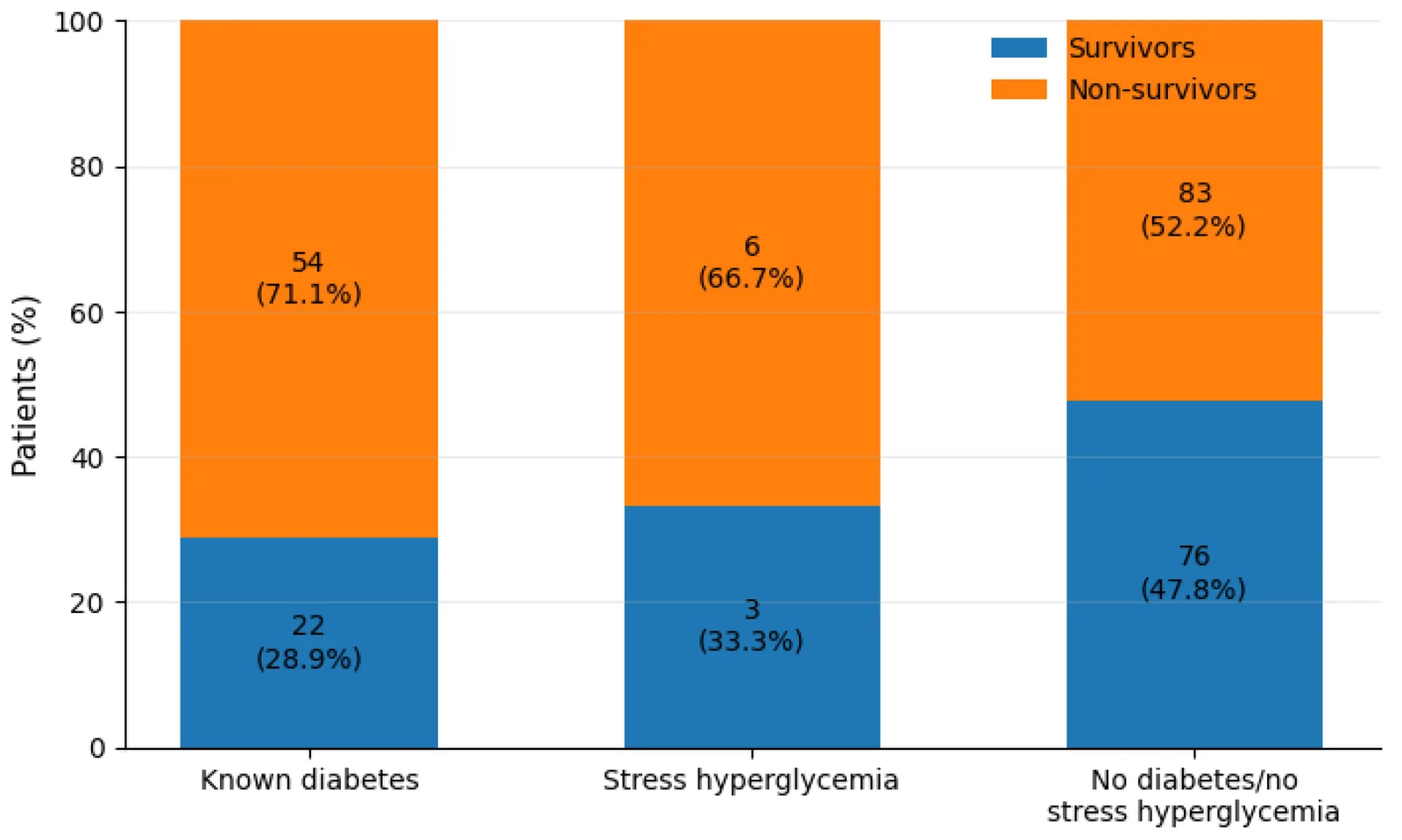
In-hospital mortality according to metabolic status. Distribution of survivors and non-survivors according to metabolic status: known diabetes mellitus, stress hyperglycemia, and no diabetes/no stress hyperglycemia.

Admission glucose, mean glucose, glycemic standard deviation, severe hyperglycemia, intravenous insulin requirement, and glycemic control differed significantly across the metabolic groups. The coefficient of glycemic variability did not differ significantly (*p* = 0.091). Because the stress hyperglycemia group included only nine patients, these between-group comparisons should be considered exploratory (Table 1 and Figure 1).

### 3.2. Association Between Glycemic Dysregulation, Mortality, and ICU Morbidity

Overall, 143 patients (58.6%) died during hospitalization, including 139 ICU deaths and four deaths after transfer to the general ward. Non-survivors were significantly older than survivors (71.05 ± 12.81 vs. 62.11 ± 15.41 years, *p* < 0.001).

Non-survivors had significantly higher admission glucose, mean glucose, glycemic standard deviation, and coefficient of glycemic variation than survivors (all *p* ≤ 0.004). Hyperglycemia > 140 mg/dL, hyperglycemia > 180 mg/dL, poor glycemic control, and intravenous insulin therapy were all associated with higher in-hospital mortality, whereas hypoglycemia < 70 mg/dL was not significantly associated with mortality.

Regarding ICU morbidity, patients with hyperglycemia > 180 mg/dL had a greater need for inotropic support (*p* < 0.001) and a longer hospital stay (*p* = 0.010). Although hypoglycemia was not associated with mortality, it was associated with a longer hospitalization (*p* = 0.045). Non-survivors also had lower GCS scores (*p* = 0.001), while SOFA scores did not differ significantly in the subset of patients with available data.

Overall, hyperglycemia, poor glycemic control, and increased glycemic variability were significantly associated with in-hospital mortality. Severe hyperglycemia (>180 mg/dL) was additionally associated with greater ICU morbidity, supporting its clinical relevance as a marker of adverse outcome (Table 2).

**Figure 2:**
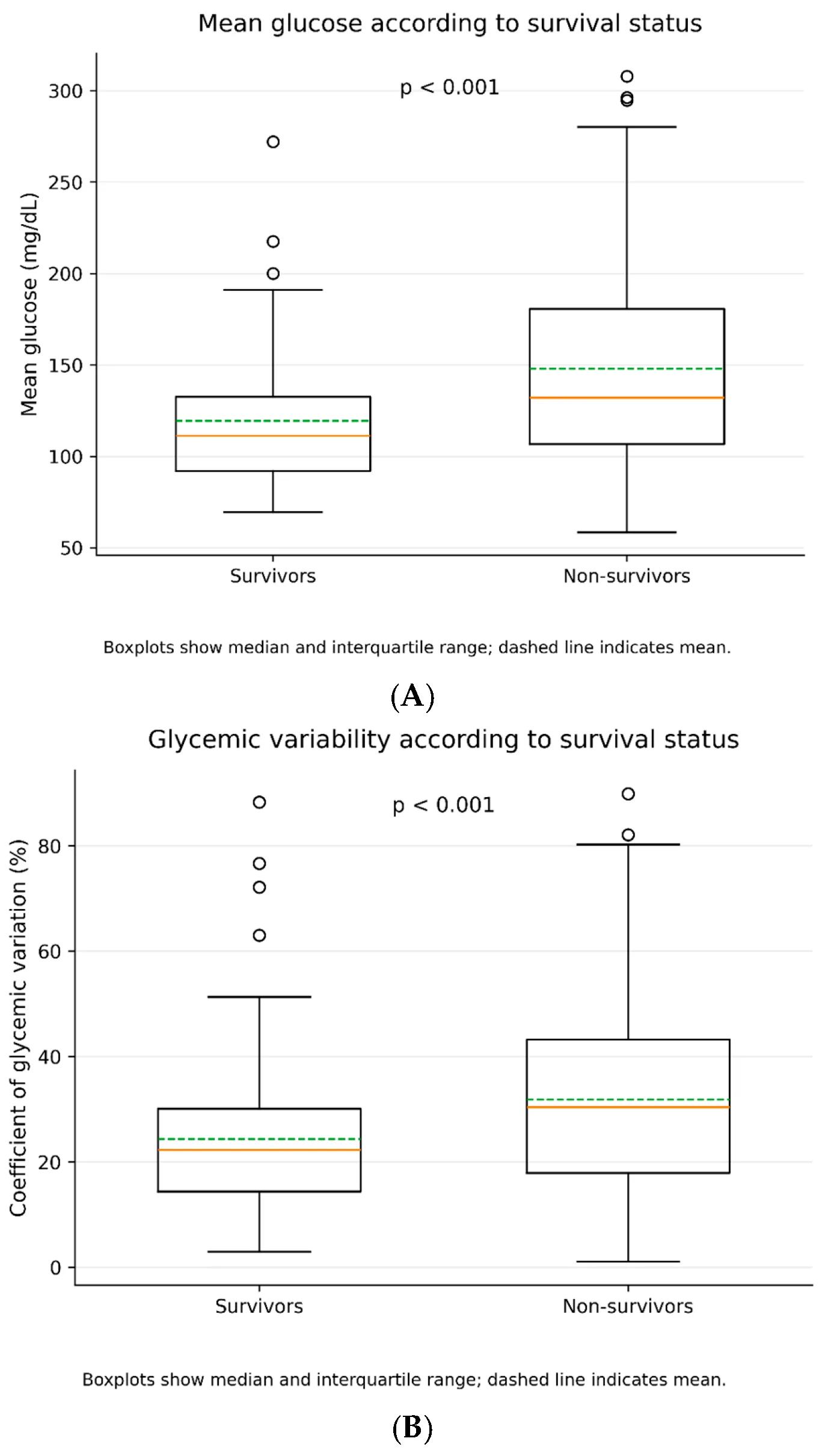
Glycemic burden and glycemic variability according to survival status. (**A**) Mean glucose levels during ICU hospitalization in survivors and non-survivors. (**B**) Coefficient of glycemic variability in survivors and non-survivors. Boxplots show median and interquartile range; dashed lines indicate mean values. Non-survivors had significantly higher mean glucose and glycemic variability compared with survivors.

Based on these findings, subsequent analyses focused specifically on the prognostic role of glycemic variability, particularly the coefficient of glycemic variability, in relation to mortality and adverse ICU outcomes (Figure 2).

### 3.3. Prognostic Relevance of Glycemic Variability in Critically Ill Patients

The median coefficient of glycemic variation (CV) was 25.60% (IQR: 16.44–36.43%). Using the predefined threshold of CV > 36%, 65 patients (26.6%) were classified as having high glycemic variability.

Patients with high glycemic variability had significantly higher mean glucose levels and glycemic standard deviation than those with lower variability (both *p* < 0.001), whereas age and admission glucose did not differ significantly.

High glycemic variability was significantly associated with in-hospital mortality (76.9% vs. 52.0%, *p* < 0.001). In univariate analysis, CV > 36% was associated with a threefold higher odds of mortality (OR = 3.08, 95% CI: 1.61–5.89), while each 10% increase in CV increased the odds of death by 36% (OR = 1.36, 95% CI: 1.13–1.62; *p* = 0.001). Mortality also increased progressively across CV tertiles (*p* = 0.003).

Patients with high CV more frequently experienced hyperglycemia > 140 mg/dL, hyperglycemia > 180 mg/dL, hypoglycemia < 70 mg/dL, and required intravenous insulin therapy more often (all *p* ≤ 0.012). Good glycemic control was significantly less frequent in this group (*p* < 0.001). Although ICU complications were numerically more common, no significant differences were observed for mechanical ventilation, inotropic support, hemodialysis, length of hospital stay, GCS, or SOFA score.

CV correlated moderately with mean glucose (Spearman’s ρ = 0.366, *p* < 0.001) and strongly with glycemic standard deviation (ρ = 0.910, *p* < 0.001), but not with age, admission glucose, hospital stay, GCS, or SOFA score.

Overall, these findings indicate that increased glycemic variability was associated with greater metabolic instability and higher in-hospital mortality, supporting its value as a dynamic prognostic marker in critically ill patients (Table 3).

**Figure 3:**
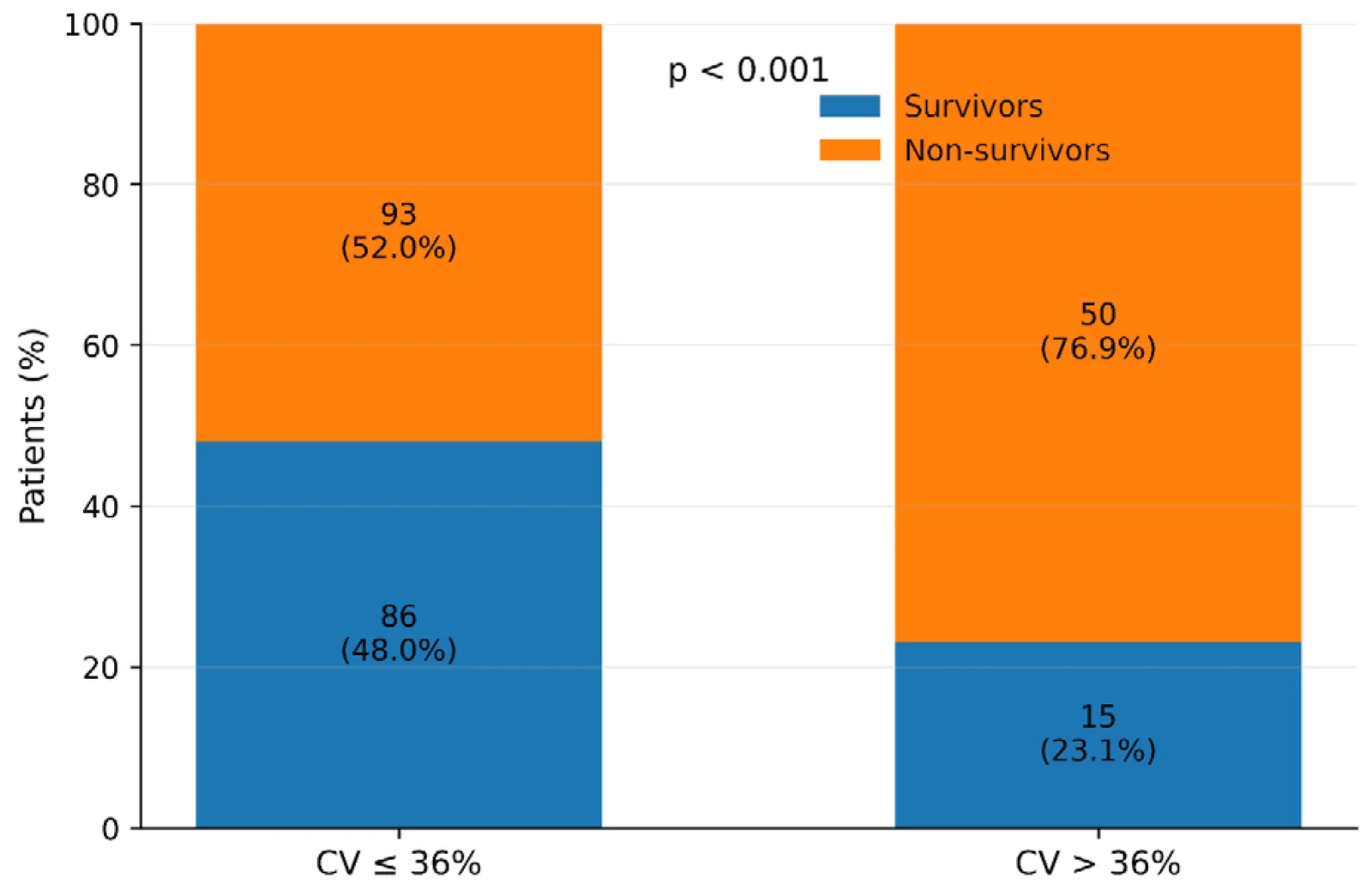
In-hospital mortality according to glycemic variability category. Stacked percentage bar chart showing the distribution of survivors and non-survivors according to glycemic variability category, defined by a CV threshold of 36%. Patients with high glycemic variability (CV > 36%) had a significantly higher in-hospital mortality rate compared with those with lower variability. In-hospital mortality included deaths occurring in the ICU and deaths after transfer to the general ward.

Given the strong unadjusted association between glycemic variability and mortality, the next analysis evaluated whether glycemic parameters remained associated with mortality after adjustment for demographic and clinical variables (Figure 3).

### 3.4. Multivariable Analysis of Factors Associated with In-Hospital Mortality

Multivariable logistic regression was performed to identify factors independently associated with in-hospital mortality. Variables were selected based on clinical relevance and univariate analyses.

In the primary model, age and mean glucose remained independently associated with mortality. Each 10-year increase in age increased the odds of death by 54% (OR = 1.54, 95% CI: 1.21–1.97; *p* = 0.001), while each 10 mg/dL increase in mean glucose increased mortality odds by 16% (OR = 1.16, 95% CI: 1.06–1.28; *p* = 0.002). Glycemic variability, diabetes status, sex, admission type, comorbidities, and hypoglycemia were not independently associated with mortality.

In the alternative model, where hyperglycemia > 180 mg/dL replaced mean glucose, glycemic variability became independently associated with mortality (OR = 1.39 per 10% increase, 95% CI: 1.12–1.73; *p* = 0.002), together with age (OR = 1.46, 95% CI: 1.16–1.83; *p* = 0.001) and medical admission (OR = 2.10, 95% CI: 1.04–4.23; *p* = 0.039). Hyperglycemia > 180 mg/dL was not independently associated with mortality after adjustment.

Sensitivity analysis including patients with available GCS data yielded similar findings. Age, mean glucose, and GCS remained independently associated with mortality, whereas glycemic variability was no longer significant.

The primary model showed acceptable discrimination (AUC = 0.782, 95% CI: 0.723–0.839), comparable to the alternative model (AUC = 0.758, 95% CI: 0.695–0.817), with no significant difference between AUCs (*p* = 0.087). Both models demonstrated good calibration according to the Hosmer–Lemeshow test.

**Figure 4:**
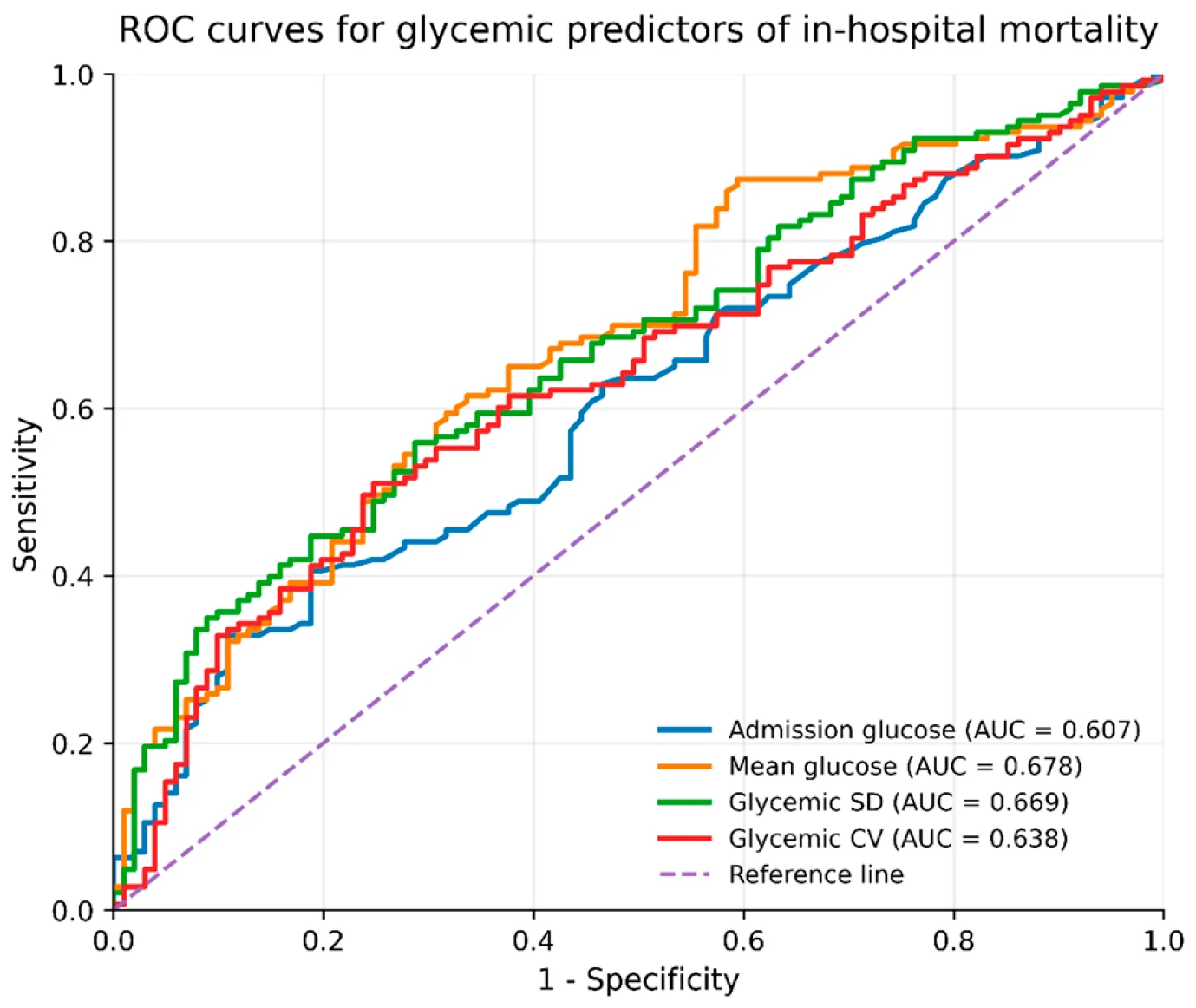
Receiver operating characteristic curves for glycemic predictors of in-hospital mortality. ROC curves evaluating the discriminatory performance of admission glucose, mean glucose during hospitalization, glycemic standard deviation, and coefficient of glycemic variability for in-hospital mortality. In-hospital mortality included deaths occurring in the ICU and deaths after transfer to the general ward. Mean glucose showed the highest discriminatory performance among the analyzed glycemic predictors.

Overall, higher mean glucose and older age were independently associated with in-hospital mortality, while glycemic variability remained independently associated with mortality only in the model excluding mean glucose (Table 4 and Figure 4).

Taken together, the results demonstrate that glycemic dysregulation was highly prevalent among critically ill patients and was closely associated with adverse clinical outcomes. Hyperglycemia, poor glycemic control, and increased glycemic variability were all significantly related to mortality in unadjusted analyses. In multivariable models, mean glucose remained the most robust glycemic predictor of in-hospital mortality, while glycemic variability retained prognostic relevance when analyzed independently from continuous glycemic burden. These findings support the clinical importance of dynamic glucose monitoring in critically ill patients, beyond isolated admission glucose values or diabetic status alone.

## 4. Discussion

The present prospective observational study evaluated the relationship between glycemic status, glycemic variability, ICU morbidity, and in-hospital mortality in a cohort of 244 critically ill diabetic and non-diabetic patients. The main findings were that glycemic dysregulation was highly prevalent, hyperglycemia was strongly associated with adverse outcomes, and increased glycemic variability identified a subgroup of patients with substantially higher mortality. In multivariable analysis, mean glucose remained the most robust glycemic predictor of in-hospital mortality, while the coefficient of glycemic variability retained prognostic relevance when analyzed independently from continuous glycemic burden. These findings support the concept that glucose dynamics during ICU stay provide clinically relevant prognostic information beyond diabetic status or admission glucose alone.

Compared with previous ICU studies focusing primarily on isolated glycemic parameters, the present prospective study simultaneously evaluated multiple complementary glycemic metrics in diabetic and non-diabetic critically ill patients and directly compared the prognostic performance of mean glucose and glycemic variability within multivariable models. This comprehensive approach provides additional insight into the relative contribution of static versus dynamic measures of dysglycemia.

A first important observation was the high frequency of hyperglycemia in the study population. Nearly three-quarters of the patients had at least one glucose value > 140 mg/dL, and more than one-quarter had glucose values > 180 mg/dL. Because only nine patients fulfilled the criteria for stress hyperglycemia, comparisons involving this subgroup should be interpreted cautiously and considered exploratory. This is consistent with the pathophysiological response to critical illness, in which acute inflammation, catecholamine release, cortisol excess, insulin resistance, altered hepatic glucose production, and therapeutic interventions such as vasopressors, corticosteroids, nutritional support, and dextrose-containing fluids may contribute to stress-related hyperglycemia. Importantly, hyperglycemia was not restricted to patients with known diabetes mellitus, indicating that acute metabolic dysregulation is a frequent phenomenon in critically ill patients regardless of previous diabetic status [10,11,12].

In this cohort, patients who died had significantly higher admission glucose, higher mean glucose during hospitalization, higher glycemic standard deviation, and higher coefficient of glycemic variability compared with survivors. These results suggest that both the magnitude and the instability of glucose exposure are clinically relevant. Admission glucose reflects the initial metabolic response to acute illness, but mean glucose and variability better capture the cumulative glycemic burden during ICU stay. The fact that mean glucose remained independently associated with mortality after adjustment for age, sex, diabetes status, admission type, comorbidities, and hypoglycemia suggests that sustained glycemic burden is not merely a descriptive marker, but may represent an important prognostic dimension in critically ill patients [13,14,15].

The association between severe hyperglycemia and adverse clinical evolution was particularly evident for the >180 mg/dL threshold. Patients exceeding this threshold had higher mortality, greater need for inotropic support, and longer hospitalization. This finding is clinically important because 180 mg/dL is commonly used as a treatment threshold in ICU glycemic management. In the present study, however, hyperglycemia > 180 mg/dL did not remain independently associated with mortality when glycemic variability was included in the alternative multivariable model. This suggests that isolated hyperglycemic thresholds may be less informative than the overall dynamic pattern of glucose fluctuation. In clinical practice, a patient with repeated oscillations between hyperglycemia and hypoglycemia may have a higher metabolic risk than a patient with stable moderate hyperglycemia [16,17,18].

Glycemic variability was one of the most relevant findings of this study. Patients with CV > 36% had a markedly higher mortality rate than those with lower glycemic variability. Moreover, mortality increased progressively across tertiles of glycemic variability, supporting a dose–response pattern. High variability was also associated with higher rates of both hyperglycemia and hypoglycemia, greater need for intravenous insulin therapy, and lower probability of achieving good glycemic control. These findings indicate that the CV integrates multiple aspects of dysglycemia, including hyperglycemic burden, hypoglycemic exposure, and instability of glucose regulation. Therefore, CV may be a practical and clinically interpretable marker for identifying patients with difficult glycemic control in ICU settings [19,20,21,22].

The prognostic role of glycemic variability may be explained by several mechanisms. Acute glucose fluctuations may amplify oxidative stress, endothelial dysfunction, inflammatory activation, mitochondrial injury, and immune dysregulation. In critically ill patients, these mechanisms may aggravate organ dysfunction and reduce physiological reserve. Moreover, high glycemic variability may also reflect the severity of underlying illness, unstable nutritional intake, inconsistent insulin sensitivity, renal or hepatic dysfunction, sepsis, or fluctuating hemodynamic status. Therefore, glycemic variability should not be interpreted only as a therapeutic failure, but also as a marker of systemic instability and critical illness complexity [23,24,25].

Recent studies have also highlighted the potential prognostic value of emerging glycemic indices such as the hemoglobin glycation index (HGI) and the stress hyperglycemia ratio (SHR). Because HbA1c measurements were not systematically available, these indices could not be calculated in the present study. Future investigations incorporating HGI and SHR may further improve risk stratification in critically ill patients [22].

The comparison between diabetic patients, patients with stress hyperglycemia, and patients without diabetes or stress hyperglycemia showed that metabolic status significantly influenced glucose profiles and treatment requirements. Patients with known diabetes and those with stress hyperglycemia had higher admission glucose, higher mean glucose, greater glycemic standard deviation, more frequent severe hyperglycemia, and higher need for intravenous insulin therapy. However, diabetes mellitus itself was not an independent predictor of mortality in multivariable analysis. This finding suggests that acute glycemic behavior during ICU stay may be more prognostically informative than diabetic status alone. In other words, the dynamic metabolic response to critical illness appears to be more relevant than the mere presence of pre-existing diabetes [26,27].

The stress hyperglycemia subgroup deserves particular attention, although it was small. These patients had the highest mean glucose values and the highest coefficient of glycemic variability, suggesting a particularly unstable metabolic phenotype. However, because only nine patients were classified as having stress hyperglycemia, these results should be interpreted as exploratory. Because HbA1c measurements were not systematically available, some patients classified as having stress hyperglycemia may have had previously undiagnosed diabetes. Therefore, this subgroup should be interpreted cautiously, and future studies should incorporate HbA1c or post-discharge metabolic evaluation to distinguish true stress hyperglycemia from previously unrecognized diabetes [28,29].

Hypoglycemia < 70 mg/dL was frequent in this cohort, affecting approximately one-quarter of patients. Although hypoglycemia was associated with numerically higher mortality, the association did not reach statistical significance in the unadjusted analysis. However, hypoglycemia was associated with longer hospitalization, suggesting that it may reflect prolonged metabolic instability, severe illness, nutritional interruptions, renal dysfunction, or the complexity of insulin therapy. From a managerial perspective, this finding remains important because prevention of hypoglycemia is a central safety objective in ICU glycemic protocols. Avoiding hypoglycemia should therefore be considered as important as preventing uncontrolled hyperglycemia [30,31,32].

The clinical severity of the cohort was high, as reflected by the need for mechanical ventilation in most patients, frequent ICU complications, and substantial in-hospital mortality. This may explain why several clinical variables showed strong associations with mortality and why glycemic markers should be interpreted in the broader context of organ dysfunction and critical illness severity. GCS remained independently associated with mortality in the subgroup with available data, while SOFA was available only for a limited number of patients and therefore could not be used as a robust adjustment variable in the main model. This represents an important limitation, because severity scores are essential for differentiating whether dysglycemia is an independent prognostic factor or mainly a marker of illness severity [33].

The findings of this study have relevant managerial implications for ICU practice. First, glucose monitoring should not be limited to admission values or isolated hyperglycemic thresholds. Instead, ICU teams should evaluate mean glucose, hypoglycemic events, and glycemic variability over time. Second, patients with CV > 36%, repeated glucose values > 180 mg/dL, or alternating hyperglycemic and hypoglycemic episodes should be considered metabolically unstable and may require closer monitoring, protocol-based insulin adjustment, and multidisciplinary reassessment of nutrition, renal function, infection status, and medication exposure. Third, a standardized ICU glycemic management protocol should aim not only to lower glucose values, but also to maintain stable glucose control while minimizing hypoglycemia [34].

These results support the need for a balanced glycemic management strategy in critically ill patients. Very strict glucose control is no longer considered appropriate for most ICU patients because it increases the risk of hypoglycemia and may worsen outcomes. Instead, contemporary critical care practice favors moderate control, usually targeting 140–180 mg/dL in most critically ill patients once insulin therapy is initiated. The present findings are consistent with this approach and further suggest that future protocols should include glycemic variability as an additional quality and safety indicator [35].

This study has several limitations. First, it was conducted in a single ICU cohort, which may limit generalizability. Second, although the prospective design strengthens data collection, the observational nature of the study does not allow for causal inference. Third, SOFA score was available only for a subset of patients, limiting the ability to fully adjust for illness severity in the main multivariable model. Fourth, HbA1c measurements were not systematically available; therefore, patients classified as having stress hyperglycemia may have included individuals with previously unrecognized diabetes, limiting precise metabolic classification. Fifth, the number of patients with stress hyperglycemia was small, and this subgroup should therefore be interpreted cautiously. Finally, glycemic measurements were obtained as part of routine clinical care, which may have introduced variability in measurement frequency according to patient severity. We acknowledge that residual confounding related to illness severity cannot be excluded.

Despite these limitations, the study provides clinically relevant evidence that glycemic dysregulation is common and prognostically meaningful in critically ill diabetic and non-diabetic patients. The integration of mean glucose, severe hyperglycemia, hypoglycemia, and glycemic variability offers a more complete picture of metabolic risk than diabetic status alone. From a managerial perspective, the results support the implementation of structured ICU glycemic protocols that incorporate dynamic glucose assessment, prevention of hypoglycemia, and systematic identification of patients with high glycemic variability. The small number of patients classified as stress hyperglycemia limited statistical power for subgroup analyses. Residual confounding related to illness severity cannot be excluded. Glucose measurement frequency may have differed between patients according to clinical condition, potentially influencing estimates of glycemic variability.

In conclusion, this study demonstrates that glycemic dysregulation is strongly associated with adverse outcomes in critically ill patients. Mean glucose was the most robust independent glycemic predictor of in-hospital mortality, while glycemic variability identified a subgroup of patients with marked metabolic instability and increased mortality risk. These findings support the clinical and managerial importance of dynamic glucose monitoring and protocol-based glycemic management in ICU patients, regardless of pre-existing diabetic status.

## 5. Conclusions

In this prospective observational study, glycemic dysregulation was frequent among critically ill diabetic and non-diabetic patients and was significantly associated with adverse outcomes. Higher mean glucose levels and increased glycemic variability were related to greater in-hospital mortality, while severe hyperglycemia and poor glycemic control were associated with increased ICU morbidity.

Mean glucose emerged as the most robust glycemic predictor of mortality, whereas the coefficient of glycemic variability identified patients with marked metabolic instability. These findings suggest that dynamic glucose monitoring may provide additional prognostic information beyond diabetic status or admission glucose alone.

From a clinical and managerial perspective, ICU glycemic management should focus not only on treating hyperglycemia, but also on minimizing excessive glucose fluctuations and preventing hypoglycemia through structured, protocol-based monitoring and individualized therapeutic adjustment.

## Figures and Tables

**Table 1:** Baseline demographic, clinical, and glycemic characteristics stratified by metabolic status.

Variable	Total Cohort, *n* = 244	Known Diabetes, *n* = 76	Stress Hyperglycemia, *n* = 9	No Diabetes/No Stress Hyperglycemia, *n* = 159	*p*-Value
Age, years	67.35 ± 14.60	70.00 ± 11.51	66.56 ± 14.82	66.13 ± 15.77	0.159
Male sex	160 (65.6%)	50 (65.8%)	6 (67%)	104 (65.4%)	0.996
Urban residence	92 (37.7%)	30 (39.5%)	5 (56%)	57 (35.8%)	0.459
Presence of comorbidities	200 (82.0%)	71 (93.4%)	7 (78%)	122 (76.7%)	0.007
Medical admission	186 (76.2%)	64 (84.2%)	4 (44%)	118 (74.2%)	0.018
Admission glucose, mg/dL	154.64 ± 76.21	207.08 ± 97.96	148.78 ± 28.02	129.91 ± 48.97	<0.001
Mean glucose, mg/dL	136.05 ± 48.37	166.89 ± 51.35	191.09 ± 43.97	118.20 ± 36.03	<0.001
Glycemic SD, mg/dL	41.57 ± 32.61	52.77 ± 34.26	68.28 ± 41.55	34.71 ± 29.03	<0.001
Coefficient of glycemic variability, %	28.70 ± 16.61	30.53 ± 15.48	36.17 ± 21.94	27.41 ± 16.72	0.091
Hyperglycemia > 140 mg/dL	182 (74.6%)	69 (90.8%)	9 (100%)	104 (65.4%)	<0.001
Hyperglycemia > 180 mg/dL	67 (27.5%)	50 (65.8%)	7 (78%)	10 (6.3%)	<0.001
Hypoglycemia < 70 mg/dL	64 (26.2%)	18 (23.7%)	1 (11%)	45 (28.3%)	0.434
Intravenous insulin therapy	75 (30.7%)	55 (72.4%)	7 (78%)	13 (8.2%)	<0.001
Good glycemic control	165 (67.6%)	29 (38.2%)	1 (11%)	135 (84.9%)	<0.001
ICU complications	194 (79.5%)	66 (86.8%)	9 (100%)	119 (74.8%)	0.031
Mechanical ventilation	227 (93.0%)	74 (97.4%)	9 (100%)	144 (90.6%)	0.112
Inotropic support	24 (9.8%)	14 (18.4%)	1 (11%)	9 (5.7%)	0.009
Hemodialysis	12 (4.9%)	5 (6.6%)	1 (11%)	6 (3.8%)	0.442
Length of hospital stay, days	9.00 (6.75–14.25)	9.50 (8.00–14.25)	11.00 (6.00–14.00)	9.00 (6.00–14.50)	0.283

Values are expressed as mean ± standard deviation, median (interquartile range), or *n* (%), as appropriate. SD, standard deviation; ICU, intensive care unit. Continuous variables were compared using the Kruskal–Wallis test, and categorical variables using the chi-square test. Owing to the small stress hyperglycemia subgroup (*n* = 9), all comparisons involving this group should be interpreted as exploratory.

**Table 2:** Glycemic parameters and clinical outcomes according to survival status.

Variable	Survivors *n* = 101	Non-Survivors *n* = 143	*p*-Value
Age, years	62.11 ± 15.41	71.05 ± 12.81	<0.001
Admission glucose, mg/dL	135.14 ± 54.61	168.42 ± 85.89	0.004
Mean glucose, mg/dL	119.35 ± 36.15	147.85 ± 52.39	<0.001
Glycemic SD, mg/dL	30.31 ± 23.24	49.52 ± 35.86	<0.001
Coefficient of glycemic variability, %	24.34 ± 14.78	31.78 ± 17.18	<0.001
Hyperglycemia > 140 mg/dL	63 (62.4%)	119 (83.2%)	<0.001
Hyperglycemia > 180 mg/dL	18 (17.8%)	49 (34.3%)	0.005
Hypoglycemia < 70 mg/dL	22 (21.8%)	42 (29.4%)	0.184
Intravenous insulin therapy	22 (21.8%)	53 (37.1%)	0.011
Good glycemic control	80 (79.2%)	85 (59.4%)	0.001
ICU complications	51 (50.5%)	143 (100.0%)	<0.001
Mechanical ventilation	85 (84.2%)	142 (99.3%)	<0.001
Inotropic support	3 (3.0%)	21 (14.7%)	0.002
Hemodialysis	4 (4.0%)	8 (5.6%)	0.561
Length of hospitalization, days	8.00 (6.00–11.00)	10.00 (7.00–17.00)	0.002
GCS score	7.44 ± 4.23	5.21 ± 3.12	0.001
SOFA score	7.47 ± 2.91	7.90 ± 3.49	0.872

Values are expressed as mean ± standard deviation, median with interquartile range, or *n* (%), as appropriate. SD = standard deviation; ICU = intensive care unit; GCS = Glasgow Coma Scale; SOFA = Sequential Organ Failure Assessment. Mortality included both ICU deaths and deaths occurring after transfer to the general ward.

**Table 3:** Clinical and glycemic characteristics according to glycemic variability.

Variable	CV ≤ 36% *n* = 179	CV > 36% *n* = 65	*p*-Value
Age, years	67.11 ± 14.30	68.02 ± 15.48	0.637
Admission glucose, mg/dL	153.50 ± 73.50	157.80 ± 83.76	0.926
Mean glucose, mg/dL	126.87 ± 42.23	161.34 ± 55.12	<0.001
Glycemic SD, mg/dL	27.02 ± 15.83	81.65 ± 33.43	<0.001
Coefficient of glycemic variability, %	20.73 ± 8.51	50.67 ± 13.34	<0.001
Hyperglycemia > 140 mg/dL	120 (67.0%)	62 (95.4%)	<0.001
Hyperglycemia > 180 mg/dL	40 (22.3%)	27 (41.5%)	0.003
Hypoglycemia < 70 mg/dL	37 (20.7%)	27 (41.5%)	0.001
Intravenous insulin therapy	47 (26.3%)	28 (43.1%)	0.012
Good glycemic control	135 (75.4%)	30 (46.2%)	<0.001
In-hospital mortality	93 (52.0%)	50 (76.9%)	<0.001
ICU complications	137 (76.5%)	57 (87.7%)	0.056
Mechanical ventilation	164 (91.6%)	63 (96.9%)	0.253
Inotropic support	18 (10.1%)	6 (9.2%)	0.848
Hemodialysis	10 (5.6%)	2 (3.1%)	0.524
Length of hospitalization, days	9.00 (6.50–15.50)	9.00 (7.00–13.00)	0.601
GCS score	5.00 (3.00–7.25)	4.00 (3.00–6.00)	0.133
SOFA score	7.50 (6.00–10.00)	6.50 (5.75–10.00)	0.391

Values are expressed as mean ± standard deviation, median with interquartile range, or *n* (%), as appropriate. CV = coefficient of glycemic variability; SD = standard deviation; ICU = intensive care unit; GCS = Glasgow Coma Scale; SOFA = Sequential Organ Failure Assessment.

**Table 4:** Multivariable logistic regression models for in-hospital mortality.

Predictor	Model 1: Mean Glucose Model OR (95% CI)	*p*-Value	Model 2: Glycemic Variability Model OR (95% CI)	*p*-Value
Age, per 10-year increase	1.54 (1.21–1.97)	0.001	1.46 (1.16–1.83)	0.001
Male sex	0.70 (0.38–1.32)	0.271	0.65 (0.36–1.21)	0.174
Diabetes mellitus	0.91 (0.44–1.90)	0.805	1.32 (0.61–2.82)	0.481
Surgical/postoperative admission	1.68 (0.82–3.45)	0.159	2.10 (1.04–4.23)	0.039
Presence of comorbidities	1.49 (0.63–3.53)	0.370	1.56 (0.67–3.63)	0.302
Mean glucose, per 10 mg/dL increase	1.16 (1.06–1.28)	0.002	—	—
Hyperglycemia > 180 mg/dL	—	—	1.49 (0.65–3.44)	0.347
Coefficient of glycemic variability, per 10% increase	1.20 (0.95–1.51)	0.133	1.39 (1.12–1.73)	0.002
Hypoglycemia < 70 mg/dL	1.89 (0.87–4.12)	0.109	1.14 (0.57–2.30)	0.711

Model 1: AUC = 0.782 (95% CI: 0.723–0.839), overall classification accuracy = 71.7%. Model 2: AUC = 0.758 (95% CI: 0.695–0.817), overall classification accuracy = 70.5%. The difference between the two AUCs was not statistically significant (*p* = 0.087). OR = odds ratio; CI = confidence interval; AUC = area under the receiver operating characteristic curve. In-hospital mortality included deaths occurring in the ICU and deaths after transfer to the general ward. Mean glucose and hyperglycemia > 180 mg/dL were analyzed in separate models to reduce overlap between continuous and categorical measures of glycemic burden.

## Data Availability

The original contributions presented in this study are included in the article. Further inquiries can be directed to the corresponding authors.

## Abbreviations

The following abbreviations are used in this manuscript:

Abbreviation	Definition
AUC	Area under the receiver operating characteristic curve
CI	Confidence interval
CV	Coefficient of variation
DM	Diabetes mellitus
GCS	Glasgow Coma Scale
HbA1c	Glycated hemoglobin
ICU	Intensive care unit
IQR	Interquartile range
IV	Intravenous
OR	Odds ratio
ROC	Receiver operating characteristic
SD	Standard deviation
SOFA	Sequential Organ Failure Assessment
SPSS	Statistical Package for the Social Sciences
